# Anxiety Symptoms in 74+ Community-Dwelling Elderly: Associations with Physical Morbidity, Depression and Alcohol Consumption

**DOI:** 10.1371/journal.pone.0089859

**Published:** 2014-02-26

**Authors:** Martina Forlani, Monica Morri, Martino Belvederi Murri, Virginia Bernabei, Francesca Moretti, Tobias Attili, Anna Biondini, Diana De Ronchi, Anna Rita Atti

**Affiliations:** 1 Department of Biomedical and NeuroMotor Sciences - Psychiatry, University of Bologna, Bologna, Italy; 2 Department of Neuroscience, Division of Psychiatry, University of Parma, Parma, Italy; Nathan Kline Institute and New York University School of Medicine, United States of America

## Abstract

**Objective:**

Anxiety among community-dwelling older adults has not been studied sufficiently. The aims of this cross-sectional population-based study were to estimate the point prevalence of clinically relevant anxiety symptoms and to describe their socio-demographic and clinical features, with particular focus on the association with somatic illnesses.

**Methods:**

Three-hundred-sixty-six non-demented older adults (mean age 83.7±6.2, range 74–99 years) from the Faenza Project (Northern Italy) were assessed using the Cambridge Mental Disorders of the Elderly Examination-Revised (CAMDEX-R) and the Geriatric Anxiety Inventory short form (GAI-sf). Multi-adjusted regression analyses were used to estimate Odds Ratio (OR) and 95% Confidence Intervals (95% CI).

**Results:**

Clinically relevant anxiety symptoms occurred in one out of five participants (point prevalence 21.0%) and were significantly associated with depression (OR 5.6 per rank; 95% CI: 3.1–10.1), physical morbidity (OR 3.5 per illness; 95% CI: 1.0–11.9) and female gender (OR 2.8; 95% CI: 1.4–5.5). Further, there were significant associations with a consumption of alcohol exceeding 1 alcoholic unit/day.

**Conclusions:**

Anxiety symptoms are very common in older subjects, especially when medically ill. Depression and alcohol consumption often co-occur with late-life anxiety symptoms, thus requiring special attention in daily clinical practice.

## Introduction

After age 65, an estimated 25% of older adults – currently around 8.6 million people worldwide - experience a mental disorder [1]. The prevalence of anxiety in community samples of older adults is especially high, ranging between 15 and 52.3%, when sub-threshold anxiety symptoms were included [2] and among women it is associated with increased mortality risk and cardiovascular mortality [3].

Frequently, anxiety disorders occur in comorbidity with other conditions [4] and the associations with chronic medical conditions and depressive disorders seem to be significant [5]–[8] but little is known about these associations after age 74. Perhaps this lack of evidence on anxiety in old-old age is due to intrinsic diagnostic challenges: in fact, discriminating symptoms due to medical conditions from physical symptoms of anxiety can be particularly problematic [9]. For example, hypertension, hyperthyroidism, hypoglycemia, mitral valve prolapse, Alzheimer’s disease, and cancer can present anxiety-like symptoms, and alcohol consumption might be used as self-medication for anxiety. Furthermore, excessive and uncontrollable worrying and common symptoms of anxiety are often associated with motor tension, vigilance and scanning. Physical symptoms like weakness, tension and sleep reduction can have organic cause while the loss of concentration might be attributed to age and cognitive impairment. In addition, older adults often deny or minimise their worries and try to justify themselves during the clinical interview. On the clinician’s side, anxiety is sometimes considered as a para-physiological characteristic of older adults, therefore is often underestimated and undertreated [10]. Conversely, the impact of anxiety in late-life is extensively being associated with significant functional limitations and impaired quality of life [11]. Also, according to patients, anxiety is the most disturbing condition associated with chronic diseases [12]. Despite the high impact on everyday life, limited literature data are currently available on the features related to anxiety among community-dwelling older adults, especially among community dwelling persons above age 74.

Hence, the main purpose of our study was to estimate the prevalence of anxiety symptoms in a group of Italian elderly people above 74 years of age. Second, we aimed to identify which socio-demographic and clinical features were associated with late-life anxiety symptoms. Our working hypotheses were that anxiety symptoms (i) are highly prevalent in persons affected by physical morbidity, (ii) often co-occur with depression, and (iii) are associated with alcohol consumption.

## Materials and Methods

### Ethics Statement

The participants first received a letter with the explanation and aims of the study and then were contacted by telephone in order to obtain initial oral consent and to book an interview. Written informed consent was obtained from all subjects after the procedures had been fully explained during the visit and before starting the interview. If, however, a person was severely cognitively impaired, a proxy (usually a close family member) was asked for written consent.

The study was approved by the Ethical Committee of the Local Health Authority of Ravenna, Ravenna, Italy.

### Participants

We analyzed data from a study on affective and cognitive effects of ageing set in Faenza, an affluent city located in northern Italy (1992-ongoing) [13]–[15]. Briefly, on January 1^st^, 2006 a randomly compiled sample of 773 individuals age 75+ were selected for cross-sectional re-evaluation. Of them, 71 (9.2%) could not be reached while 702 were invited to take part in the study; of these, 462 (65.8%) accepted (mean age 85.09±6.86 years; 53.2% of women). In order to investigate a cognitively well-functioning population, we excluded 88 subjects (19.0%) suffering from dementia [16]. Data concerning anxiety symptoms were not available for 8 cases, leaving a total of 366 subjects for this study.

### Instruments and Diagnoses

Participants were administered the Cambridge Mental Disorders of the Elderly Examination-Revised (CAMDEX-R) [17], [18], a diagnostic schedule with the following main sections: 1) a structured psychiatric interview incorporating questions regarding present mental disorder and past personal and family history; 2) an objective evaluation of a broad range of cognitive functions; 3) a standardized schedule to record observations on the present mental state, appearance and demeanor; 4) a structured interview with a relative or informant who could provide information on the respondent’s present state, changes in personality and activities of daily living; 5) a brief physical examination (including neurological items) with a review of various laboratory findings and medication in use, when applicable.

The Geriatric Anxiety Inventory short form (GAI-sf) [9], [19], [20] was used to detect the presence of anxiety symptoms in the elderly, using a cut-off score of three or more. This instrument demonstrated a sensitivity of 75%, a specificity of 87%, and a positive predictive value of 86% for anxiety in older subjects. In this population, GAI-sf score was not related to age, MMSE score, level of education or perceived income adequacy. Internal consistency was high (Cronbach’s *α = *0.81) and concurrent validity against the State-Trait Anxiety Inventory was good (rs = 0.48, p*<*0.001).

Diagnoses related to physical conditions were based on the International Classification of Diseases, tenth edition (ICD-10) [21]. The following conditions were included: anemia (D50–64), diabetes (E10–14), hypertension (I10), coronary heart disease (CHD) (I20), cerebrovascular diseases (I64), Parkinson’s Disease (G20). We defined physical morbidity as the coexistence of multiple chronic diseases [22]. A categorical variable (no disease; 1; 2 and 3 or more) was created and having no disease was used as reference category.

Depression and its severity were diagnosed according to ICD-10 criteria (F32) [20], [14]. Severity was evaluated based on the number of symptoms as mild (F32.0), moderate (F32.1) and severe (F32.2).

The Mini Mental State Examination (MMSE) and the Cambridge Cognitive Examination (CAM-Cog), were used to evaluate global cognitive functions. Scores were entered as continuous variables.

### Socio-demographic Features

Age was used both as a continuous and as a categorical variable. We created four age strata: ≤80 years; 81–85 years; 86–90 years; ≥91 years. Education was defined according to the number of years of schooling as a categorical variable (illiterate and incomplete primary school; primary school or more). Marital status was codified as never married, married and no longer married (widowed and divorced). The current socio-economic status (SES), as self-reported variables, was defined in three classes, low, medium and high. Participant’s current drinking habits were also explored and the quantity of each beverage drunk was converted into unit/day. We assumed an alcoholic unit as a glass of wine (125 ml), a can of beer (330 ml) and a small glass of hard-liquor (40 ml) [23]. Both men and women were assigned to four groups of current alcohol consumption: less than 1 alcohol unit/day, 1 alcohol unit/day (used as reference category), 2 alcohol units/day and more than 2 alcohol units/day.

### Statistical Analyses

All the Statistical analyses were performed using SPSS (Statistical Package for Social Science).

Chi-square test and Student’s t test for independent samples were used respectively to compare frequencies and means. Logistic regression analyses were used to estimate Odds Ratios (OR) and 95% Confidence Intervals (95% CI). First we tested the univariate associations between anxiety symptoms and each variable of interest, then we included all significant predictors in the model to account for their possible confounding role. All analyses were first implemented using the presence/absence of the above mentioned variables, and then in terms of severity levels: no depression *vs* mild or moderate-severe, no physical morbidity *vs* 1, 2, 3 or more diseases.

## Results

**Figure 1:**
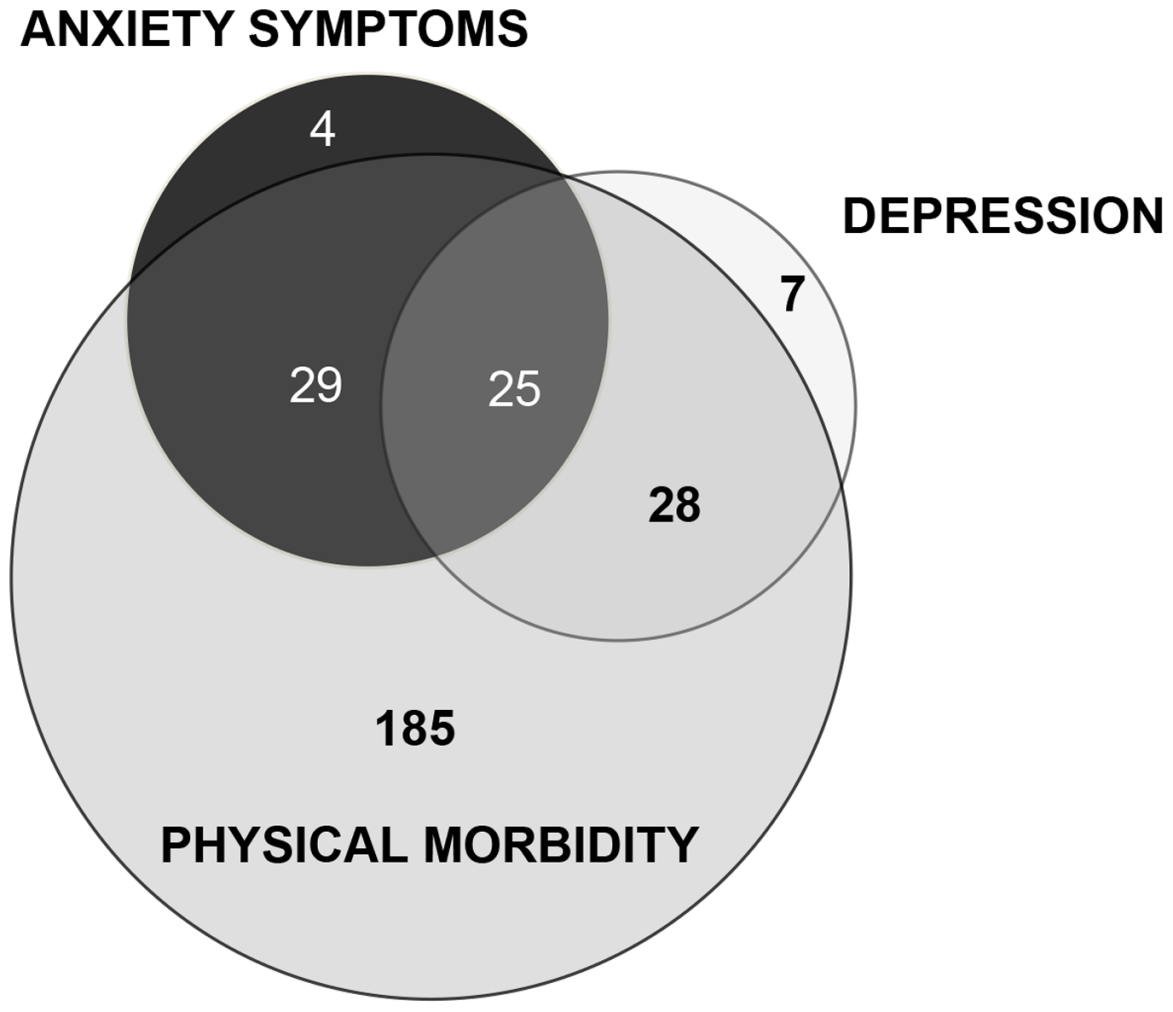
Absolute number of subjects with overlapping anxiety symptoms, depression and physical morbidity. As showed in the figure, anxiety symptoms co-occur with depression and physical morbidity in 25 older adults, 43,1% of the group with anxiety. Physical diseases are associated with anxiety symptoms in 29 subjects and with depression in 28 subjects. Anxiety symptoms were defined by the Geriatric Anxiety Inventory short form, using a cut-off score of three or more; depression was diagnosed according to ICD 10 Criteria; physical morbidity was defined as having at least one physical disease among anemia, diabetes, hypertension, coronary heart disease (CHD), cerebrovascular diseases, Parkinson’s Disease. Subjects with missing data for anxiety symptoms or depression or physical morbidity were excluded, leaving a total of 278 subjects.

The demographic and health-related characteristics of the sample of 366 older adults are shown in table 1: approximately 50% of the participants were married; the majority of the participants reported a medium level of SES, while 25,2% suffered from depression. With regards to physical health, only 17.4% reported no physical illnesses. More than 80% of the population had at least one chronic disease and 9.9% more than three diseases. Anxiety symptoms occurred in 77 participants (21,04%), mostly women (63.6%). Subjects with anxiety symptoms were two years younger than those who were not affected (mean age 82.4±5.4 vs. 84±6,4, p = 0.053). The proportion of individuals with anxiety symptoms decreased with increasing age: after 90 years they were only 7,8%. We didn’t find associations between anxiety symptoms and MMSE, CAM-Cog, marital status, years of education and SES. Instead, anxiety symptoms often co-occurred with depression and physical morbidity: among subjects with more than three chronic diseases (n = 9), one out of two suffered also of anxiety symptoms. Figure 1 illustrates the overlap between anxiety symptoms, depression and physical morbidity. Only 5.2% of the elderly (n = 4) had isolated anxiety symptoms, whereas 32,5% (n = 25) suffered also from depression and physical morbidity. Psychiatric symptoms (either anxiety and/or depression) occurred in 44,3% of those with at least one somatic disease.

**Table 1:** Socio-demographic and clinical characteristics of the sample and subjects with anxiety symptoms.

			Sample	Anxiety symptoms		Test Value, df, *p* *
			N = 366	NO (N = 289)	YES (N = 77)	
**Gender**	Males	*N (%)*	182 (49.7)	154 (53.3)	28 (36.4)	*X* ^2^ = 6.966, df = 1, *p* = 0.008
**Age, years**		*Mean (SD)*	83.7 (±6.2)	84 (±6.4)	82.4 (±5.4)	t = 1.939, df = 364, *p* = 0.053
**Age, groups**	≤80 y	*N (%)*	136 (37.1)	104 (36.0)	32 (41.5)	*X* ^2^ = 8.445, df = 3, *p* = 0.038
	81–85 y	*N (%)*	95 (26.0)	74 (25.6)	21 (27.3)	
	86–90 y	*N (%)*	66 (18.0)	48 (16.6)	18 (23.4)	
	≥91 y	*N (%)*	69 (18.9)	63 (21.8)	6 (7.8)	
**Education^α^**	<5 y	*N (%)*	144 (39.3)	115 (40.1)	29 (37.7)	*X* ^2^ = 0.147, df = 1, *p* = 0.701
	≥5 y	*N (%)*	220 (60.7)	172 (59.9)	48 (62.3)	
**MMSE**		*Mean (SD)*	26.4 (±3.7)	26.4 (±3.7)	26.4 (±3.8)	t = 0.099, df = 364, *p* = 0.712
**CAMCOG**		*Mean (SD)*	78.7(±15.4)	78.6(±15.5)	79.5(±14.9)	t = −0.482, df = 352, *p* = 0.125
**Marital status** ^β^	Never married	*N (%)*	22 (6.1)	17 (5.9)	5 (6.8)	*X* ^2^ = 1.648, df = 2, *p* = 0.439
	Married	*N (%)*	178 (49.4)	137 (47.9)	41 (55.4)	
	No longer married	*N (%)*	160 (44.5)	132 (46.2)	28 (37.8)	
**SES^γ^**	Low	*N (%)*	38 (10.5)	25 (8.7)	13 (16.9)	*X* ^2^ = 4.971, df = 2, *p* = 0.083
	Medium	*N (%)*	319 (87.9)	257 (89.9)	62 (80.5)	
	High	*N (%)*	6 (1.6)	4 (1.4)	2 (2.6)	
**Depression^δ^**	No	*N (%)*	273 (74.8)	238 (82.4)	35 (46.0)	*X* ^2^ = 42.940, df = 2, *p*<0.001
	Mild	*N (%)*	60 (16.4)	35 (12.1)	25 (32.9)	
	Moderate-Severe	*N (%)*	32 (8.8)	16 (5.5)	16 (21.1)	
**Physical morbidity^φ^**	None	*N (%)*	63 (17.4)	57 (19.8)	6 (7.9)	*X* ^2^ = 6.150, df = 2, *p* = 0.052
	1	*N (%)*	156 (43)	119 (41.5)	37 (48.7)	
	2	*N (%)*	108 (29.7)	86 (30.0)	22 (28.9)	
	≥3	*N (%)*	36 (9.9)	25 (8.7)	11 (14.5)	
**Alcohol, units/day^δ^**	<1	*N (%)*	150 (41.1)	114 (39.6)	36 (46.7)	*X* ^2^ = 8.021, df = 3, *p* = 0.046
	1	*N (%)*	106 (29.0)	93 (32.3)	13 (16.9)	
	2	*N (%)*	88 (24.1)	67 (23.3)	21 (27.3)	
	>2	*N (%)*	21 (5.8)	14 (4.8)	7 (9.1)	

*Comparison between persons with and without anxiety symptoms; *X^2^* = Pearson Chi-square test, t = Student’s t test for independent samples, df = degree of freedom; MMSE (Mini Mental State Examination); CAM-Cog (Cambridge Cognitive Examination); SSE (Socio-economic status). Missing data ^α^N = 2; ^β^N = 6; ^γ^N = 3; ^δ^N = 1; ^φ^N = 3.

Table 2 shows the results of multivariate regression analyses: female gender, having more than two physical morbidities, depression and drinking more than one alcohol unit per day were all associated with anxiety symptoms; instead, the subjects that were more than 90 years old, had a lower probability to suffer from anxiety symptoms. After adjustment for gender, age, physical morbidity and alcohol consumption, anxiety symptoms were associated with depression severity in a “dose-dependent” manner: OR (95% CI) = 4.832 (2.456–9.508) for mild depression and OR (95% CI) = 7.283 (3.087–17.182) for moderate or severe depression (*p*<0.001).

**Table 2:** Odds Ratios (OR) and 95% Confidence Intervals (95% CI) for anxiety symptoms in relation to female gender, age, physical morbidity, depression and alcohol consumption, estimated by multivariate logistic regression analyses.

		Model 1		Model 2		Model 3		Model 4		Model 5	
		OR (CI 95%)	*p*	OR (CI 95%)	*p*	OR (CI 95%)	*p*	OR (CI 95%)	*p*	OR (CI 95%)	*p*
**Gender**	Female	1.937(1.142–3.285)	0.014	2.302 (1.338–3.961)	0.003	2.282 (1.315–3.960)	0.003	2.440 (1.352–4.404)	0.003	2.818 (1.439–5.519)	0.003
**Age,** **groups**	≤80 y	−		1 (Ref.)		1 (Ref.)		1 (Ref.)		1 (Ref.)	
	81–85 y	−	−	0.937 (0.492–1.785)	0.843	0.876 (0.455–1.687)	0.693	0.772 (0.382–1.562)	0.472	0.801 (0.392–1.634)	0.542
	86–90 y	−	−	1.144 (0.568–2.304)	0.707	1.043 (0.511–2.128)	0.909	1.055 (0.488–2.278)	0.892	1.229 (0.554–2.725)	0.612
	≥91 y	−	−	0.254 (0.098–0.658)	0.005	0.257 (0.098–0.677)	0.006	0.338 (0.124–0.923)	0.034	0.373 (0.133–1.047)	0.055
**Physical** **morbidity**	None	−	−	−	−	1 (Ref.)		1 (Ref.)		1 (Ref.)	
	1	−	−	−	−	2.579 (1.007–6.602)	0.048	2.487 (0.923–6.697)	0.072	2.281 (0.823–6.319)	0.113
	2	−	−	−	−	2.526 (0.939–6.792)	0.066	2.840 (0.995–8.108)	0.051	2.494 (0.849–67.327)	0.096
	≥3	−	−	−	−	3.627 (1.170–11.243)	0.026	3.711 (1.119–12.305)	0.032	3.525 (1.041–11.934)	0.043
**Depression**	No	−	−	−	−	−	−	1 (Ref.)		1 (Ref.)	
	Mild	−	−	−	−	−	−	4.813 (2.469–9.380)	<0.001	4.832 (2.456–9.508)	<0.001
	Moderate-Severe	−	−	−	−	−	−	7.164 (3.098–16.563)	<0.001	7.283 (3.087–17.182)	<0.001
**Alcohol,** **units/day**	1	−	−	−	−	−	−	−	−	1 (Ref.)	
	<1	−	−	−	−	−	−	−	−	1.908 (0.866–4.205)	0.109
	2	−	−	−	−	−	−	−	−	2.483 (1.030–5.988)	0.043
	>2	−	−	−	−	−	−	−	−	4.242 (1.189–15.142)	0.026

Subjects with missing value for at least one variable were excluded, leaving 355 subjects for each model.

Model 1, gender; Model 2, gender and age; Model 3, gender, age and physical morbidity; Model 4, gender, age, physical morbidity and depression;

Model 5, gender, age, physical morbidity, depression and alcohol consumption.

Regarding alcohol use, no association was found between abstainers and anxiety symptoms; instead there was an increased probability for subjects to report anxiety symptoms with the increasing number of alcohol units/day drunk compared to people in the reference category: OR (95% CI) = 2.483 (1.030–5.988) for 2 alcohol units/day and OR (95% CI) = 4.242 (1.189–15.142) for more than 2 alcohol units/day.

## Discussion

We aimed to investigate the prevalence and aspects of anxiety symptoms in a representative population of Italians, age 74+. We found a high prevalence of anxiety symptoms, approximately 21%. This estimate lies between the prevalence of generalized anxiety disorder according to DSM criteria (which ranges between 1.2% and 15%) and the prevalence of anxious symptoms (which reaches up to 52.3%) [2], [4]. Differences in population composition and instruments used to record anxiety symptoms might explain such a discrepancy. Our study shows a higher prevalence of anxiety symptoms in females than in males (26% *vs* 15%) and a lower prevalence with increasing age. These findings confirm previous observations in literature [24], [25], claiming that women could be more prone to anxiety since they display a higher degree of interpersonal sensitivity and emotional involvement when facing life or adversities. A consistent body of literature suggests that anxiety disorders occurrence is lower in old age and the results of our study confirm this pattern among the oldest subjects [8], [26]–[29].

However, whether the lower the prevalence of anxiety with aging is true or due to a healthy survivors effect, remains to be established.

We found strong correlations between anxiety symptoms and depression, as is well known in literature [5], [6], [10], [25], [30]–[33]. It has been demonstrated that, among older persons, “anxious depression” may be a common clinical presentation [8]. In a dimensional view, anxiety and depression may be positioned on a continuum, or viewed as alternative manifestations of a common underlying diathesis; pure anxiety often progresses to depression or co-morbid anxiety-depression [34]. It has also been shown that depression and anxiety share the same factors of vulnerability (namely, high neuroticism and low mastery), but stressful life events might have differing origins: the onset of depression is predicted by loss-related events (i.e. of a partner or a relative), while anxiety is predicted mostly by interpersonal conflict or the incidence of illness of the subjects’ family or friends [25].

In our study the proportion of participants with anxiety symptoms but not depression is 56,9% and 87,9% of them had at least one physical morbidity. In summary, findings in literature to date are still somewhat conflicting: although anxiety often co-occurs with depression, a significant proportion does not. Mehta *et al.* [35] found that although anxiety occurred in 43% of their sample who were depressed, 15% of their non-depressed participants also reported anxiety symptoms. Beekman *et al.* [5] reported that 25% of older people with anxiety suffered from major depression, while 50% of those with depression had symptoms of anxiety.

Last, the association between anxiety symptoms and physical morbidity deserves comment, as several mechanisms could underlie this finding. First, psychological problems (e.g., anxiety) can increase the vulnerability to physical disease. For example, an older adult who is afraid of falling often restricts his/her mobility in order to reduce the risk of falls, and that results in reduced exercise and increased risk for physical disease associated with being sedentary (e.g., heart disease, pneumonia, diabetes). Second, physical diseases can cause psychological symptoms: a diagnosis of heart disease may lead to anxiety or concern about experiencing a heart attack during physical activity. Third, a variety of medical conditions manifest physical symptoms of both motor tension and autonomic arousal that can be difficult to distinguish from an anxiety disorder. Fourth, medication side effects can imitate psychological symptoms. For example, medications that are commonly prescribed for older adults (e.g., antidepressants, antihypertensive) might have side effects similar to anxiety symptoms such as insomnia and decreased concentration [27]. Last, anxiety and physical illness can occur simultaneously.

In literature, the correlation between alcohol use and anxiety is unclear. Our data suggest a significant association between anxiety symptoms and the consumption of alcohol. In older men and women, these findings are not unusual since moderate regular alcohol consumption is associated with improved mood, increased quality of life and helps protect against cognitive decline [36]. The cross-sectional association between alcohol use and anxiety symptoms found in our elderly population might be related with the social role of alcohol consumption or with self-medication.

The results of this study must be viewed in light of a few limitations: the most significant is the unicentric and cross-sectional study design, which does not allow us to assess the direction of causal relationships. Further, although based on a psychometrically sound instrument [20] the assessment of anxiety was based on few dichotomous items of CAMDEX, and this may have contributed to inflate the detected prevalence.

Last, we were unable to detect more specific anxiety disorders, like agoraphobia or GAD as was done in other studies [3,37].

Despite these limitations, this study adds valuable information on late-life anxiety symptoms suggesting that the majority of subjects with anxiety symptoms might also suffer from physical illnesses or use alcohol. The most recent evidence on the clinical management of late-life anxiety does not provide the clinician with sufficient indications for its diagnosis and management in persons with co-morbid physical illnesses or considerable alcohol use. Further, research should extend our knowledge about this issue and the mechanisms underlying the association between anxiety and physical illnesses.

## Conclusion

We found that besides being commonly associated with depression, late-life anxiety is often comorbid with physical disorders, in a dose-response fashion, and with alcohol consumption. Clinicians should be aware of these factors whilst in the process of recognition and treatment of anxiety.
